# Degradation of Host Sphingomyelin Is Essential for *Leishmania* Virulence

**DOI:** 10.1371/journal.ppat.1000692

**Published:** 2009-12-11

**Authors:** Ou Zhang, Mattie C. Wilson, Wei Xu, Fong-Fu Hsu, John Turk, F. Matthew Kuhlmann, Yingwei Wang, Lynn Soong, Phillip Key, Stephen M. Beverley, Kai Zhang

**Affiliations:** 1 Department of Biological Sciences, Texas Tech University, Lubbock, Texas, United States of America; 2 Department of Internal Medicine, Washington University School of Medicine, St. Louis, Missouri, United States of America; 3 Department of Microbiology and Immunology, Department of Pathology, The University of Texas Medical Branch, Galveston, Texas, United States of America; 4 Department of Molecular Microbiology, Washington University School of Medicine, St. Louis, Missouri, United States of America; Albert Einstein College of Medicine, United States of America

## Abstract

In eukaryotes, sphingolipids (SLs) are important membrane components and powerful signaling molecules. In *Leishmania*, the major group of SLs is inositol phosphorylceramide (IPC), which is common in yeast and Trypanosomatids but absent in mammals. In contrast, sphingomyelin is not synthesized by *Leishmania* but is abundant in mammals. In the promastigote stage *in vitro*, *Leishmania* use SL metabolism as a major pathway to produce ethanolamine (EtN), a metabolite essential for survival and differentiation from non-virulent procyclics to highly virulent metacyclics. To further probe SL metabolism, we identified a gene encoding a putative neutral sphingomyelinase (SMase) and/or IPC hydrolase (IPCase), designated *ISCL* (**I**nositol phospho**S**phingolipid phospholipase **C-L**ike). Despite the lack of sphingomyelin synthesis, *L. major* promastigotes exhibited a potent SMase activity which was abolished upon deletion of *ISCL*, and increased following over-expression by episomal complementation. *ISCL*-dependent activity with sphingomyelin was about 20 fold greater than that seen with IPC. Null mutants of *ISCL* (*iscl^−^*) showed modest accumulation of IPC, but grew and differentiated normally *in vitro*. Interestingly, *iscl^−^* mutants did not induce lesion pathology in the susceptible BALB/c mice, yet persisted indefinitely at low levels at the site of infection. Notably, the acute virulence of *iscl^−^* was completely restored by the expression of *ISCL* or heterologous mammalian or fungal SMases, but not by fungal proteins exhibiting only IPCase activity. Together, these findings strongly suggest that degradation of host-derived sphingomyelin plays a pivotal role in the proliferation of *Leishmania* in mammalian hosts and the manifestation of acute disease pathology.

## Author Summary

*Leishmania* are obligate intracellular parasites responsible for a spectrum of diseases in humans ranging from self-healing skin lesions to deadly visceral infections. To survive, they must downregulate the microbicidal activity and scavenge nutrients from the host. Although *Leishmania* parasites do not synthesize sphingomyelin, which is an abundant lipid in mammals, they do possess the ability to hydrolyze sphingomyelin. This neutral sphingomyelinase (SMase) activity is dependent on an *ISCL* gene, which is also required for the degradation of inositol phosphorylceramide, the dominant sphingolipid in *Leishmania*. Deletion of *ISCL* in *L. major* led to a complete loss of SMase activity but no obvious defects in growth or differentiation *in vitro*. Remarkably, null mutants of *ISCL* failed to induce any detectable pathology in mammals yet were able to persist at low levels indefinitely. Such defect was completely reversed when a functional neutral SMase was introduced into the mutant. In total, our results suggest that degradation of host-derived sphingomyelin by *Leishmania* is essential for acute virulence. Further studies will elucidate the pivotal role of sphingolipid homeostasis in the molecular interaction between *Leishmania* parasites and their mammalian hosts.

## Introduction

*Leishmania* parasites infect 10–12 million people worldwide, causing a spectrum of diseases known as leishmaniasis [1]. Transmitted by sandflies, these protozoan pathogens have tremendous negative impacts on public health worldwide, especially in developing countries [1]. During their life cycle, *Leishmania* parasites alternate between flagellate promastigotes which live in the midgut of sandfly and non-flagellate amastigotes which reside in the phagosome of mammalian macrophages. Control of *Leishmaniasis* has been hampered by the lack of a safe vaccine, limitation of frontline drugs, and the emergence of drug resistant strains [2]. To develop new and more cost effective drugs, it is necessary to understand the molecular mechanism of *Leishmania* pathogenesis. A number of surface molecules including lipophosphoglycan (LPG), glycosylinositolphospholipids (GIPLs), and the metalloprotease gp63 provide resistance to digestive enzymes, reactive oxygen species, and complement-mediated lysis [3],[4]. Other virulence factors including the LACK antigen (*Leishmania* homologue of receptors for activated C kinase) [5] and cysteine proteases play important roles in modulating host immune response [6].

In addition to these well-studied virulence factors, recent work has provided insight into the function of sphingolipids (SLs) in *Leishmania*. SLs are ubiquitous membrane components in eukaryotes with well-documented functions in general membrane physiology, raft formation, cell-to-cell recognition, and signaling [7]–[10]. Unlike mammalian cells which synthesize sphingomyelin and glycosylsphingolipids to high abundance, the majority of SLs in *Leishmania* are unmodified inositol phosphorylceramide (IPC), a class of lipids mainly found in fungi and plants [11],[12]. The functions of SLs metabolism in *L. major* were recently probed using two deletion mutants: a *spt2^−^* strain that is SL-free due to the deletion of an essential subunit gene (*SPT2*) of serine palmitoyltransferase, the first enzyme in the *de novo* synthesis of SLs (Fig. S1) [12],[13]; and a *spl^−^* strain that lacks the degradative enzyme sphingosine-1-phosphate lyase (SPL) (Fig. S1) [14]. Both mutants were fully viable and replicated normally during log phase growth; however, they died quickly in stationary phase and failed to differentiate from non-infective procyclics to infective metacyclics (metacyclogenesis) [14]. Remarkably, supplementation of ethanolamine (EtN) or phosphoethanolamine (a product of SPL, Fig. S1) completely reversed the viability and differentiation defects of both mutants [14]. EtN is likely used to synthesize plasmenylethanolamine (PLE, the dominant phosphatidylethanolamine species in *L. major*), a class of plasmalogen lipid that is highly abundant in metacyclics [12],[14]. These results indicate that a main function of SL synthesis and degradation is to generate EtN, a metabolite essential for survival and metacyclogenesis in *Leishmania* promastigotes (Fig. S1). The conversion from SLs to phosphoethanolamine could occur via two pathways: an IPC-independent route directly from sphingoid bases or ceramide, or an alternative route which requires the synthesis and degradation of IPC (Fig. S1).

Contrary to the problems encountered during metacyclogenesis, *spt2^−^* and *spl^−^* amastigotes (the non-motile forms that reside in macrophages) were morphologically normal and fully infective [14]; in addition, expression of *SPT2* (mRNAs and protein) was greatly downregulated in wild type (WT) amastigotes [12]; finally, amastigotes of WT and *spt2^−^* contained abundant amounts of IPC and PLE (similar to WT promastigotes), despite the loss of *de novo* SL synthesis and EtN production [15]. Since IPC is not found in mammals, these results imply: 1) amastigotes acquire SL metabolites from the mammalian host, either directly or through the hydrolysis of host SLs to generate metabolite; and 2) salvage of host lipids may play important roles for the survival and proliferation of *Leishmania* amastigotes. To explore the functions of SL metabolism that are independent of EtN production, it is necessary to study the enzymes that are directly involved in the uptake and degradation of host SLs in *Leishmania*.

In mammalian cells, the sphingomyelinase (SMase)-mediated sphingomyelin hydrolysis is the major pathway to produce stress-induced ceramide (Fig. S2) [16]. Ceramide is a well-studied bioactive molecule implicated in many signaling processes including apoptosis, inflammation, growth and differentiation [17]. Among the five known classes of SMases, neutral SMases have emerged as the main candidates for the production of stress-induced ceramide [16]. Although yeasts do not synthesize sphingomyelin, they produce abundant amount of glycosylated inositol phosphoceramides (glycosylated IPC) [18],[19]. The functional homologue of mammalian neutral SMase in yeasts is inositol phosphosphingolipid-phospholipase C or IPCase (Fig. S2) [20]. In *Saccharomyces cerevisiae*, the activity and localization of IPCase (ScISC1p; encoded by *ScISC1*) were regulated in a growth-dependent manner: predominantly in the endoplasmic reticulum (ER) during early log stage but associated with mitochondria in late logarithmic growth, which might lead to the activation of this enzyme by the anionic phospholipids in the mitochondrial membrane [21],[22]. Deletion of *ScISC1* resulted in a slow growth phenotype, suggesting it was required for the utilization of non-fermentable carbon sources and/or the respiration function of mitochondria [22]. The null mutant was also more sensitive to heat and high salt concentration, which seemed to be linked to the deregulation of several stress-response genes [23],[24]. In the opportunistic fungal pathogen *Cryptococcus neoformans*, *CnISC1* (which encodes the IPCase in *C. neoformans* or CnISC1p) was critical for the survival of this pathogen in macrophages and its dissemination to the brain [25]. Neither IPCase nor neutral SMase has been studied in protozoa.

In this report, we identified a single *ISCL* gene in *L. major* homologous to the neutral SMase genes in mammals and the *ISC1* genes in fungi (Fig. S3). To study its role in SL metabolism and pathogenesis, null mutants of *ISCL* were generated through targeted gene deletion and results suggest the ability to degrade sphingomyelin is essential for acute virulence in *Leishmania*.

## Results

### Identification and targeted replacement of *ISCL*

Through a BLASTp search, *ISCL* (system ID: LmjF08.0200, 1962 bp) was identified from the *L. major* genome (www.genedb.org) as the sole homologue of human neutral SMase 1 (Genbank accession #NP_003071) and *S. cerevisiae* ISC1p (Genbank accession #P40015) (Fig. S3). The predicted protein (ISCL, 653 aa) contains several conserved amino acids including Glu51, Asp116, Asp383, and His384 (Fig. S3). From studies of *Bacillus cereus* SMase [26], *Sc*ISC1p [27], and human neutral SMase 1 [28], these residues may be critical for Mg^2+^ binding, substrate recognition, and catalytic activity. In addition, ISCL possesses a P-loop motif (His115 to Lys122) which is found in phosphatases and nucleotide-binding proteins and may be essential for catalytic efficiency [27]), and two predicted transmembrane helices near the C-terminus (Ala447 to Arg466 and Trp612 to Val 634) which may tether the protein to anionic phospholipid-rich membranes [29] (Fig. S3).

**Figure 1:**
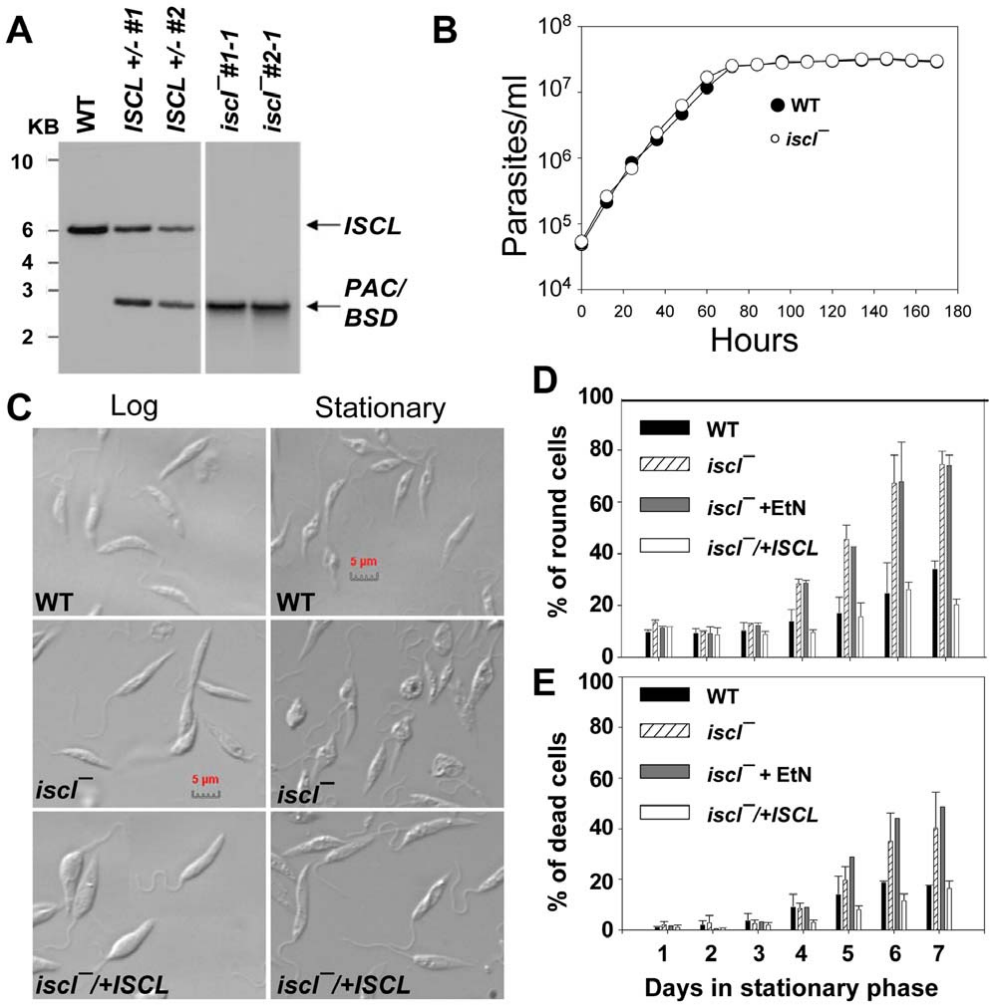
*ISCL* is not required for growth in promastigotes but plays a crucial role in cell shape and vitality in late stationary phase. (**A**) Targeted gene deletion of *ISCL*. Southern blot analysis of *L. major* wild type (WT), heterozygote clones (*ISCL*+/− #1 and 2), and homozygote clones (*iscl^−^* #1-1 and #2-1) was performed as described in ***Materials and Methods*** using a probe adjacent to the *ISCL* locus. Bands corresponding to the *ISCL* locus (6.1 Kb) and replacements with drug markers (2.8 Kb for *PAC* and 2.6 Kb for *BSD*) are indicated. (**B**) *Iscl^−^* mutants had a normal growth rate. Promastigotes were inoculated in M199 medium at 3.0×10^4^ cells/ml and culture densities were determined every 8–12 hours using a hemocytometer. (**C**)–(**E**) *Iscl^−^* mutants showed altered morphology and poor viability in late stationary phase. (**C**) Representative DIC images of log and late stationary phase (four days after reaching maximal density) parasites. After entering stationary phase, cells were analyzed daily for morphology (**D**, by microscopic observations) and viability (**E**, by flow cytometry of propidium iodide-positive cells). Experiments were performed in duplicate or triplicates and error bars represent standard deviations.

To examine the role of *ISCL* in SL degradation and virulence, null mutants were generated in *L. major* through two rounds of targeted gene replacement as previously described, since *Leishmania* are predominantly diploid [30],[31]. Southern-blot analysis confirmed the loss of both *ISCL* alleles in two candidate *iscl^−^* (*ΔISCL::PAC/ΔISCL::BSD*) clonal lines (Figs. 1A and S4). As a control, these mutants were complemented with an episome (pXG-*ISCL*) which drove the overexpression of *ISCL* (Fig. S4). The two clonal lines showed very similar phenotypes, and thus data for only clone #1-1 and its reconstituted control (*iscl^−^/+ISCL*) are presented here.

### *Iscl^−^* promastigotes were fully viable in log phase but showed altered morphology and poor viability in late stationary phase

In culture, *iscl^−^* promastigotes grew normally with a doubling time of ∼7.0 hours during logarithmic growth phase, indicating *ISCL* is not required for survival or replication in the insect stage (Fig. 1B). The *iscl^−^* mutants also attained similar densities as WT parasites in stationary phase (2.5–3.3×10^7^ cells/ml) (Fig. 1B). Interestingly, although *iscl^−^* promastigotes exhibited normal morphology in log phase and the first 1–2 days in stationary phase (Fig. 1C–D), they became progressively less elongated in late stationary phase (Fig. 1C–D). Microscopic evaluations revealed that after 3 days in stationary phase, 30–40% of *iscl^−^* cells became round (round cells were defined as those with the length of the long axis less than twice the length of the short axis) whereas only 10–20% of WT cells were round (Fig. 1C–D). This difference became more pronounced as cultures continued to age, with 70–80% of round cells in *iscl^−^* versus 24–38% in WT after 5 days in stationary phase (Fig. 1C–D). This round phenotype is characteristic of unhealthy promastigotes, consistent with the increased percentage of dead cells as 35–49% of *iscl^−^* became permeable to propidium iodide versus 10–20% of WT after 3–4 days in stationary phase (Fig. 1E). These viability and shape defects did not arise from programmed cell death as *iscl^−^* parasites showed no characteristics of apoptosis (such as the exposure of phosphatidylserine or PtS, data not shown). Importantly, stationary phase defects were solely due to the loss of *ISCL*, as the *iscl^−^/+ISCL* and *ISCL*+/− (heterozygote) parasites showed normal viability and morphology (Fig. 1 C–E and data not shown). Finally, these viability and shape defects could not be reversed by EtN (Fig. 1D–E), which is different from the stationary phase defects exhibited by *spt2^−^* and *spl^−^* mutants [14].

### *Iscl^−^* mutants showed no apparent defects in differentiation to metacyclics

**Figure 2:**
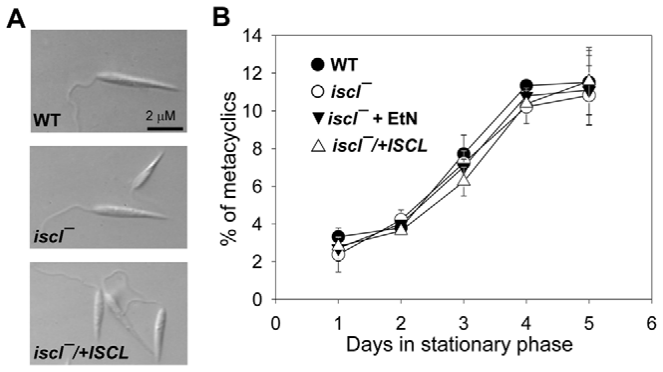
Metacyclogenesis is normal in *iscl^−^* mutants. (**A**) Representative DIC images of metacyclics purified from stationary phase cultures using the density centrifugation method. (**B**) Percentages of metacyclics were determined daily from low passage (<5) stationary cultures of WT, *iscl^−^* (grown in the presence or absence of 200 µM of EtN), and *iscl^−^/+ISCL*) using the density centrifugation method. Experiments were performed in duplicates and error bars represent standard deviations.

For *Leishmania* promastigotes, the cessation of growth in stationary phase coincides with the onset of metacyclogenesis, i.e. differentiation from non-infective procyclic forms to infective metacyclic forms [32]. Metacyclics can be distinguished from procyclics based on morphology, reactivity to lectins and monoclonal antibodies, and density gradient sedimentation [33],[34]. Since mutant parasites showed altered morphology and increased death in stationary phase, it was important to examine whether the loss of *ISCL* affected metacyclogenesis. To do so, we isolated metacyclics from stationary phase *iscl^−^* parasites after a Ficoll density gradient centrifugation [34]. These *iscl^−^* metacyclics had very similar morphology as those purified from WT and *iscl^−^/+ISCL* parasites (Fig. 2A). The percentage of *iscl^−^* metacyclics increased progressively and peaked at 10–13% after 3–4 days in stationary phase, as seen with WT and *iscl^−^/+ISCL* parasites (Fig. 2B). Similar results were observed when metacyclics were isolated using the peanut agglutination method which is based on cell surface carbohydrate and antigenic changes between metacyclics and procyclics [33] (data not shown). Together, these data suggest *ISCL* is not required for metacyclogenesis, although is critically involved in the maintenance of cell shape, and, to a lesser degree, cell viability in late stationary phase.

### *ISCL* is required for the degradation of sphingomyelin and IPC

**Figure 3:**
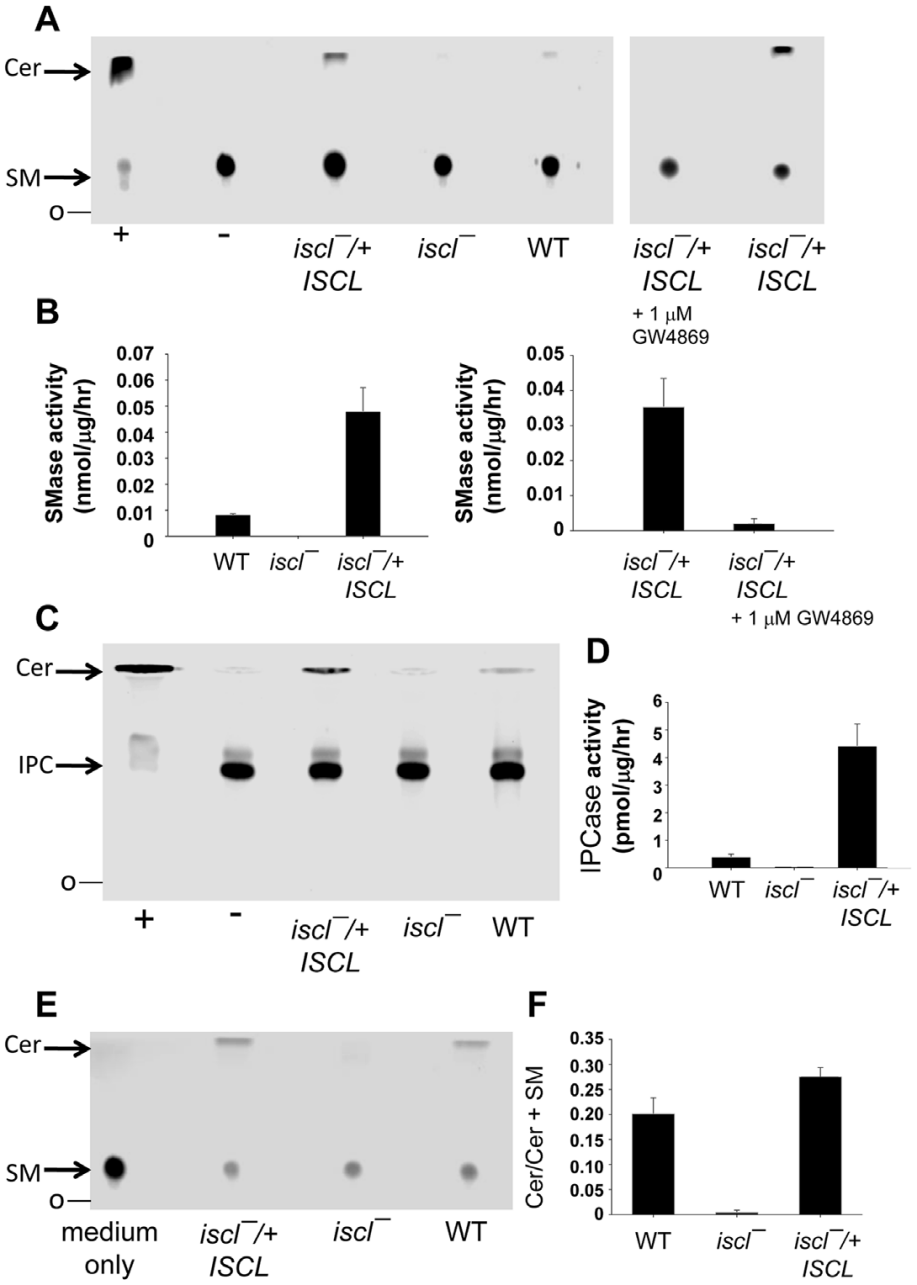
*ISCL* is required for the hydrolysis of sphingomyelin and IPC. (**A**)–(**B**) *In vitro* neutral SMase assay. Whole cell lysates from log phase promastigotes (∼40 µg of protein each) were incubated with TX100-based micelles containing NBD-labeled sphingomyelin as described in ***Materials and Methods***. Neutral SMase inhibitor GW4869 was provided as indicated (1 µM final concentration). Lipids were extracted and separated by TLC. Plates were scanned using a Storm 860 phosphoimager and the activity of SMase was normalized to nmol/µg/hour. Positive control (+): 0.1 unit of *B. cereus* SMase; negative control (−): boiled WT lysate. (**C**)–(**D**) Degradation of sphingomyelin by promastigotes *in vivo*. Parasites were cultured in the presence of NBD-labeled sphingomyelin for 48 hours and lipids were extracted and separated by TLC (“medium only” means NBD-labeled sphingomyelin was incubated in the absence of parasites for 48 hours). Ratio of ceramide/ceramide + sphingomyelin (SM) was determined using an Image Analysis Software (ImageQuant TL 7.0) and used as an indicator of sphingomyelin hydrolysis. (**E**)–(**F**) *ISCL* is required for the degradation of IPC. Whole cell lysates from log phase promastigotes (∼40 µg of protein each) were incubated with TX100-based micelles containing NBD-labeled IPC as described in ***Materials and Methods***. Lipids were extracted and separated by TLC. Plates were scanned using a Storm 860 phosphoimager and the activity of IPCase was normalized to pmol/µg/hour. Experiments were performed in duplicates and error bars represent standard deviations. **O**: origin of TLC.

Since ISCL is a homolog of mammalian neutral SMase and fungal ISC1p, we tested whether it is required for the hydrolysis of sphingomyelin, IPC, or both. Briefly, *Leishmania* lysates were incubated with Triton X100/lipid mixed micelles prepared as described by Okamoto et al. [29] with minor modifications. When a NBD-labeled C6 sphingomyelin was used as substrate with whole cell extracts from WT log phase promastigotes, ceramide (one of the degradative products of SMase) was detected by thin layer chromatography (TLC) (Fig. 3A–B). This high level of SMase activity was comparable to what was observed from mammalian and yeast whole cell lysate [35],[36]. In contrast, lysates from *iscl^−^* mutants did not induce ceramide production and were similar to the negative control made of boiled lysate from WT parasites (Fig. 3A–B), indicating *ISCL* is required for the hydrolysis of sphingomyelin *in vitro*. As expected, the complemented strain *iscl^−^/+ISCL*, predicted to have increased expression *ISCL* due to overexpression from a multicopy episomal vector, exhibited higher SMase activity (5–7 times more than WT, Fig. 3A–B; Table 1). Furthermore, we tested whether this *ISCL*-mediated SMase activity was sensitive to a specific inhibitor of mammalian neutral SMase 2, GW4869. As shown in Fig. 3A–B, at a final concentration of 1 µM, GW4869 completely shut down the degradation of sphingomyelin in *iscl^−^/+ISCL*, indicating that this ISCL-dependent *Leishmania* SMase activity is as sensitive as the murine neutral SMase 2 (IC50 around 1 µM [37]).

**Table 1:** Summary of neutral SMase and IPCase activity in *L. major* promastigote lysates.

Activity (pmol/µg/hr)	WT	*iscl^−^*	*iscl^−^*/+*ISCL*	*iscl^−^SSU::hNSM1*	*iscl^−^SSU::mNSM2*	*iscl^−^/+ ScISC1*	*iscl^−^/+ CnISC1*
SMase	7.9	<0.1	42	21	100	1.9	0.4
IPCase	0.39	<0.1	4.4	0.1	0.2	0.3	5.8

Normalized activities of neutral SMase and IPCase (average values) in log phase promastigotes were determined from whole cell extracts (Figs. 3, 5, and data not shown).

Next, we tested whether *ISCL* was required for the hydrolysis of sphingomyelin by intact promastigotes. To do so, parasites were metabolically labeled with NBD C6 sphingomyelin for 48 hours and cellular lipids were extracted and analyzed by TLC. As a control, we added NBD C6 sphingomyelin to growth medium without parasites for 48 hours and results showed very little spontaneous degradation (Fig. 3C). Both WT and *iscl^−^/+ISCL* parasites were capable of sphingomyelin uptake and degradation, whereas *iscl^−^* mutants only showed uptake without hydrolysis (Fig. 3C–D). Therefore, *ISCL* is essential for the SMase activity *in vivo*. Interestingly, *iscl^−^/+ISCL* parasites only caused ∼40% more degradation (based on quantative analysis of the ceramide/ceramide + sphingomyelin ratio) than WT parasites (Fig. 3C–D). This differs from the SMase activity data acquired from cell lysate where *iscl^−^/+ISCL* showed 5–7 times more activity than WT (Fig. 3A–B).

We next examined whether *Leishmania* ISCL possessed IPCase activity. Assays were carried out similarly to those examining sphingomyelin hydrolysis, but using Triton X100/lipid mixed micelles containing PtS and NBD-labeled C12 IPC, followed by lipid extraction and TLC. As shown in Fig. 3E and 3F, WT parasites exhibited a detectable level of IPCase activity whereas *iscl^−^* mutants failed to degrade IPC. Episomal expression of ISCL in *iscl^−^/+ISCL* led to a marked increase of IPCase activity, suggesting ISCL is involved in the IPC degradation (Fig. 3E–F). Notably, the specific activity of IPCase is 10–20 times lower than that of SMase (Table 1), suggesting sphingomyelin is the preferred substrate of ISCL.

We then examined the cellular level of IPC, ceramide, and PLE (which is synthesized from SL-derived EtN) in WT, *iscl^−^*, and *iscl^−^/+ISCL* promastigotes by electrospray ionization mass spectrometry (ESI/MS) in the negative ion mode (Table 2). Abundances of IPC (composed of d16:1/18:0-PI-Cer, d18:1/18:0-PI-Cer, and d16:1/18:0-PI-PhytoCer [12]) and PLE (composed of *p*18:0/18:2-PtE and *p*18:0/18:1-PtE [12]) were estimated through comparison with appropriate internal standards (d18:1/8:0-ceramide phosphate for IPC, d18:1/8:0-ceramide for ceramide, and *p*18:0/20:4-PtE for PLE). As summarized in Table 2, *iscl^−^* mutants contained 53–59% more IPC and 30–52% less ceramide than WT and *iscl^−^/+ISC* parasites, consistent with a role for ISCL in IPC degradation. The cellular level of PLE in *iscl^−^* mutants was very similar to WT and *iscl^−^/+ISCL* parasites (Table 2). As described before [12], *L. major* promastigotes did not contain sphingomyelin.

**Table 2:** Abundance of IPC, PLE, and ceramide in promastigotes.

Molecules/cell (×10^8^)	WT	*iscl^−^*	*iscl^−^*/+*ISCL*
IPC	2.52 ± 0.52	3.55 ± 0.36	2.41 ± 0.47
PLE	1.57 ± 0.33	1.72 ± 0.23	1.47 ± 0.42
Ceramide	2.61 ± 0.20	1.91 ± 0.07	3.41 ± 0.47

Abundances of IPC, PLE, and ceramide in stationary phase promastigotes (3 days after reaching maximal density) were determined by quantative mass spectrometry (with internal standards) as described in ***Materials and Methods***. Average values from two independent experiments are summarized with standard deviations.

### *Iscl^−^* mutants failed to induce pathology in susceptible mice but persisted for at least 7 months

**Figure 4:**
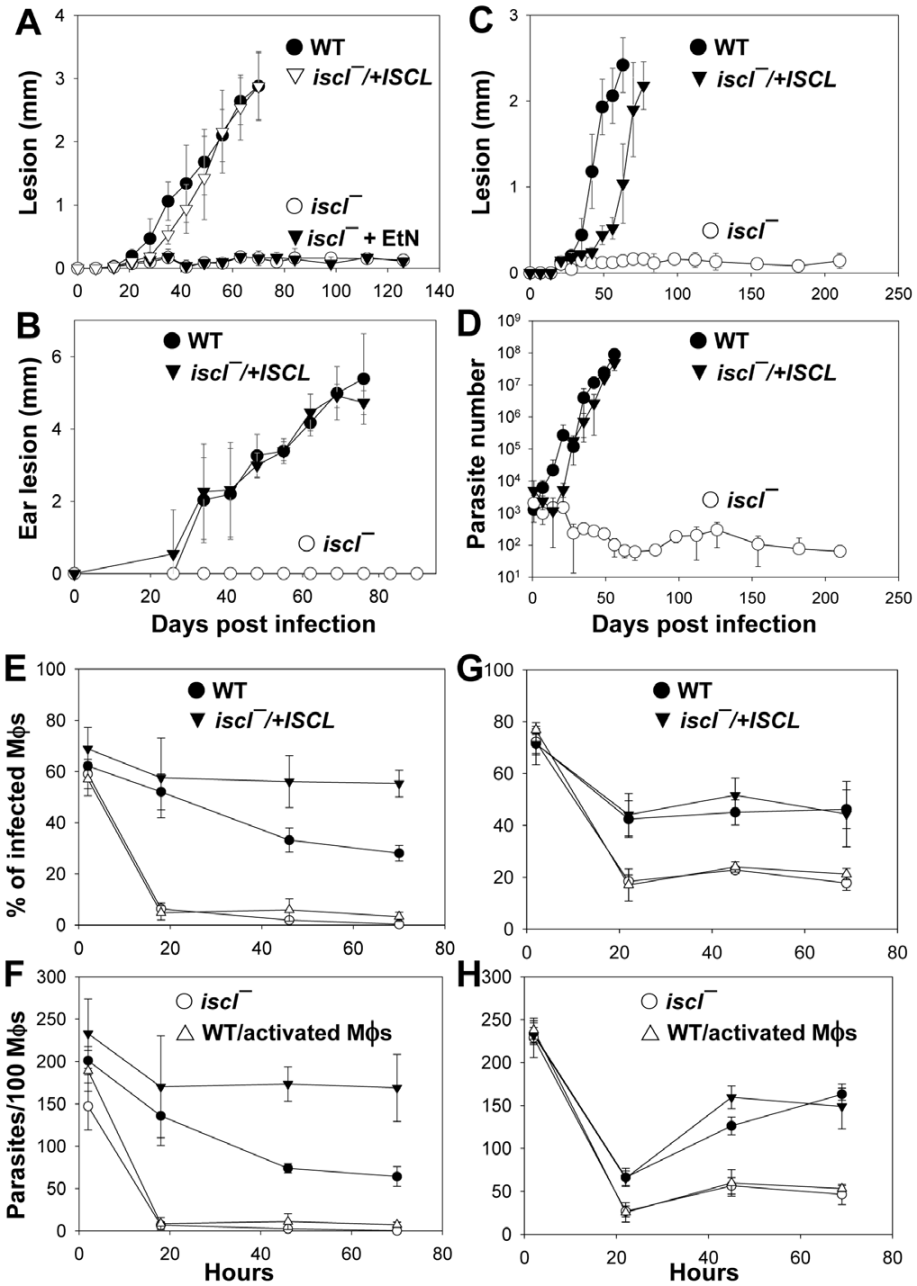
*ISCL* is essential for acute virulence but not persistence. (**A**)–(**B**) *Iscl^−^* mutants failed to induce pathology in mice. BALB/c mice were infected in the footpads (**A**) or ears (**B**) with stationary phase promastigotes and lesion sizes were measured weekly with a caliper. (**C**)–(**D**) *Iscl^−^* mutants were able to persist at low levels. BALB/c mice were infected in the footpads with purified metacyclics at 2×10^5^ parasites per mouse. Lesion sizes were measured with a caliper (**C**) and parasite numbers in infected footpads were determined by the limiting dilution assays (**D**). (**E**)–(**H**) *Iscl^−^* infection of peritoneal macrophages (Mφs). Stationary phase promastigotes were used to infect peritoneal Mφs from BALB/c mice as described [38]. Percentages of infected Mφs (**E**, **G**) and the number of parasites per 100 Mφs were recorded (**F**, **H**). Experiments were performed at 37 °C (**E**, **F**) or 33 °C (**G**, **H**). As a control, WT parasites were also used to infect activated Mφs (▵: stimulated with 100 ng/ml of LPS and 100 ng/ml of IFN-γ) to ensure these Mφs possessed microbicidal activities. Error bars represent standard deviations.

Next, we assessed the virulence of *iscl^−^* mutants in BALB/c mice, which are highly susceptible to *L. major*. In footpad and ear infections, WT, *ISCL*+/− (heterozygote), and *iscl^−^/+ISCL* parasites caused rapid progression of lesion which correlated with the increasing number of parasites in the infected tissue (Fig. 4A–D and data not shown). Remarkably, neither stationary phase promastigotes nor purified metacyclics of *iscl^−^* mutants induced detectable lesions in the footpads or ears of BALB/c mice (Fig. 4A–C). This virulence defect was not reversed by EtN (Fig. 4A), which makes *iscl^−^* clearly different from the *spt2^−^* and *spl^−^* mutants [14]. The defect in cell viability was not sufficient to cause a complete loss of virulence in *iscl^−^* because we used 3-day old stationary phase promastigotes of which 85–95% of cells were healthy as judged by PI-exclusion (Figs. 1E, 4A–B). In addition, metacyclic forms of *iscl^−^* that were morphologically normal and impermeable to propidium iodide also failed to cause pathology (Fig. 3C–D and data not shown). Despite the lack of pathology, limiting dilution assays showed *iscl^−^* mutants were able to persist at the site of infection at very low levels (50–120 parasites/footpad) for at least 7 months post infection (Fig. 4D). Therefore, *ISCL* was essential for acute virulence and pathology but not long-term persistence.

To corroborate the results obtained in mouse infections, we examined the virulence of *iscl^−^* mutants in an *in vitro* macrophage infection assay using peritoneal macrophages isolated from BALB/c mice [38]. When the infection was performed at 37 °C, *iscl^−^* mutants were able to bind and enter macrophages efficiently (as shown by the 2-hour time points in Fig. 4E–F) but these parasites failed to replicate thereafter and were quickly eliminated (Fig. 4E–F). In contrast, when the infection was done at 33 °C, *iscl^−^* mutants were able to survive in macrophages, albeit at a lower level than WT and *iscl^−^/+ISCL* parasites (2–3 times lower in both infection rate and the number of parasites/100 macrophages, Fig. 3G–H). The ability to survive better at lower temperatures could be related to the ability to survive for long periods in peripheral mouse tissues like the footpad or ear. Our results also imply *ISCL* is involved in heat tolerance as the *iscl^−^/+ISCL* parasites, which overproduced ISCL from a multicopy episome (pXG-*ISCL*), survived better than WT at 37 °C in macrophages (Fig. 4E–F). The ability to survive better at a lower temperature could be related to the ability to survive for long periods in peripheral mouse tissues like the footpad or ear.

### Heterologous expression of mammalian neutral SMases and fungal ISC1ps in *iscl^−^*

To probe the importance of SMase vs. IPCase in *Leishmania*, we expressed SL hydrolases of known specificity in the *iscl^−^* mutant. These included human neutral SMase 1 (NP_003071), murine neutral SMase2 (NM_021491.3), and the *S. cerevisiae* ISC1p, as this latter enzyme exhibits both SMase and IPCase activity [39]. Human and murine ORFs were cloned into the pIR1SAT vector and integrated into the rRNA locus (to generate *iscl^−^* SSU::hNSM1 or *iscl^−^* SSU::mNSM2, respectively) which results in high levels of expression [40]. Similarly, *ScISC1* was inserted into the multicopy episomal vector pXG and transfected into the *iscl^−^* mutant yielding *iscl^−^*/+*ScISC1*, which also yields high level of expression.

**Figure 5:**
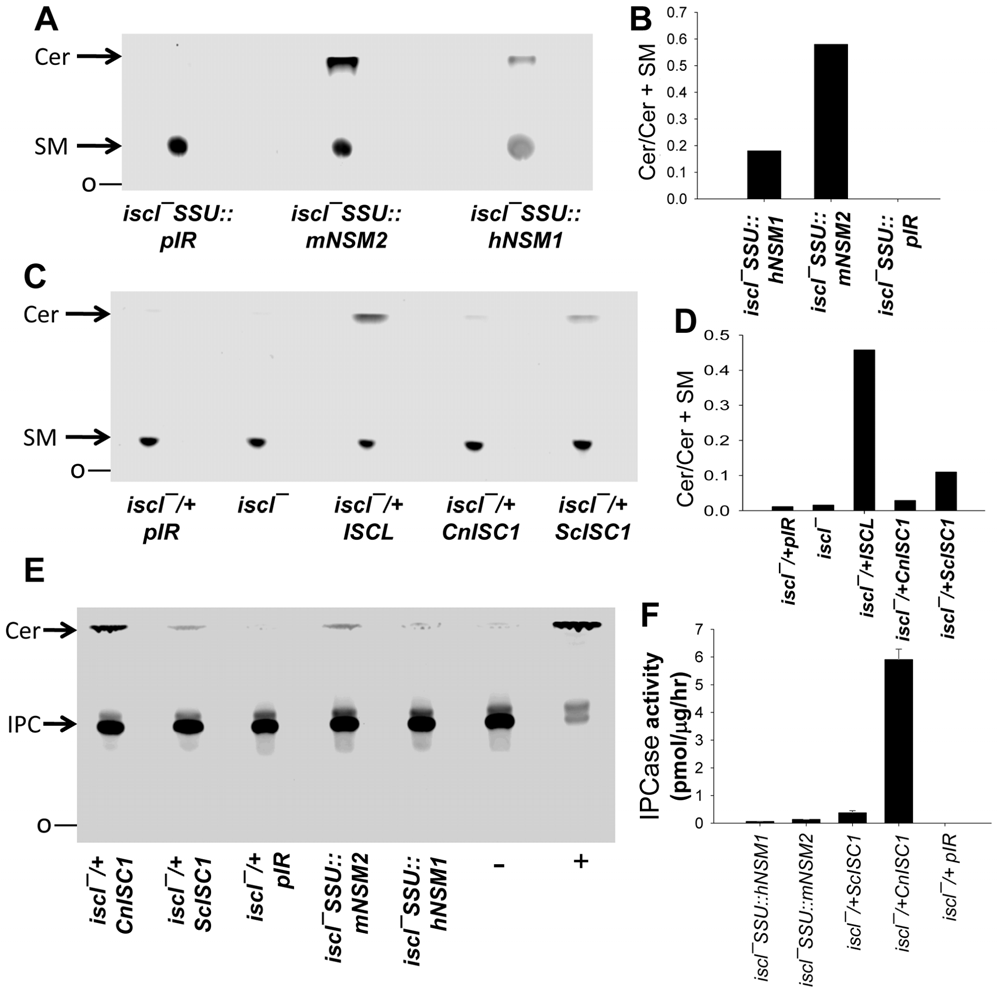
Heterologous expression of mammalian neutral SMases and fungal ISC1ps in *iscl^−^*. (**A**)–(**B**) *Iscl^−^* parasites with human neutral SMase 1 (*iscl^−^* SSU::hNSM1), murine neutral SMase 2 (*iscl^−^* SSU::mNSM2), or pIR1SAT vector only (*iscl^−^* SSU::pIR) were labeled with NBD-sphingomyelin and lipids were examined as described in Fig. 4C. (**C**)–(**D**) Similar experiments were performed using *iscl^−^* parasites transfected with pIR1SAT-*ScISC1* (*iscl^−^/+ScISC1*), pIR1SAT-*CnISC1* (*iscl^−^/+CnISC1*), and pIR1SAT vector only (*iscl^−^*/+pIR). To evaluate the degradation of NBD-sphingomyelin, ratios of ceramide/ceramide + sphingomyelin were determined using an Image Analysis Software (ImageQuant TL 7.0) and shown in **B** and **D**. (**E**) IPC degradation assay was performed using whole cell lysates from *iscl^−^* parasites transfected with hNSM1, mNSM2, *ScISC1*, or *CnISC1*; results were normalized to pmol/mg/hour (**F**). Positive control (+): 0.1 unit of *B. cereus* PI-PLC; negative control (−): boiled WT lysate. Experiments were repeated multiple times and error bars represent standard deviations.

We examined the ability of these transgenic parasites to hydrolyze sphingomyelin *in vivo* following provision of NBD C6 sphingomyelin as described above. Both human neutral SMase 1 and murine neutral SMase 2 possessed the ability to break down sphingomyelin when expressed in *iscl^−^* promastigotes (Fig. 5A and 5B); in contrast, control parasites transfected with the pIR vector alone (*iscl^−^* SSU::pIR) did not exhibit SMase activity despite maintaining sphingomyelin uptake (Fig. 5A and 5B). Similarly, expression of *ScISC1* restored the ability to degrade sphingomyelin in *iscl^−^* (Fig. 5C–D). Consistent with these *in vivo* labeling results, we were able to detect strong SMase activity in the *in vitro* assay with whole cell lysates from *iscl^−^* SSU::hNSM1, *iscl^−^* SSU::mNSM2, and *iscl^−^*/+*ScISC1*, but not from *iscl^−^* SSU::pIR (data not shown). Similar *in vitro* experiments were performed to assess whether these SMases could degrade IPC (using NBD C12 IPC as described above) when expressed heterologously in *iscl^−^* mutants. As shown in Fig. 5E–F, *ScISC1* and murine neutral SMase 2 induced modest IPC degradation, whereas the activity from human neutral SMase 1 was close to background. Similar to *L. major* ISCL, the IPCase activity exhibited by these enzymes were much lower compared to SMase activity (Table 2).

### Complementation of *iscl^−^* mutants by heterologous SMases

**Figure 6:**
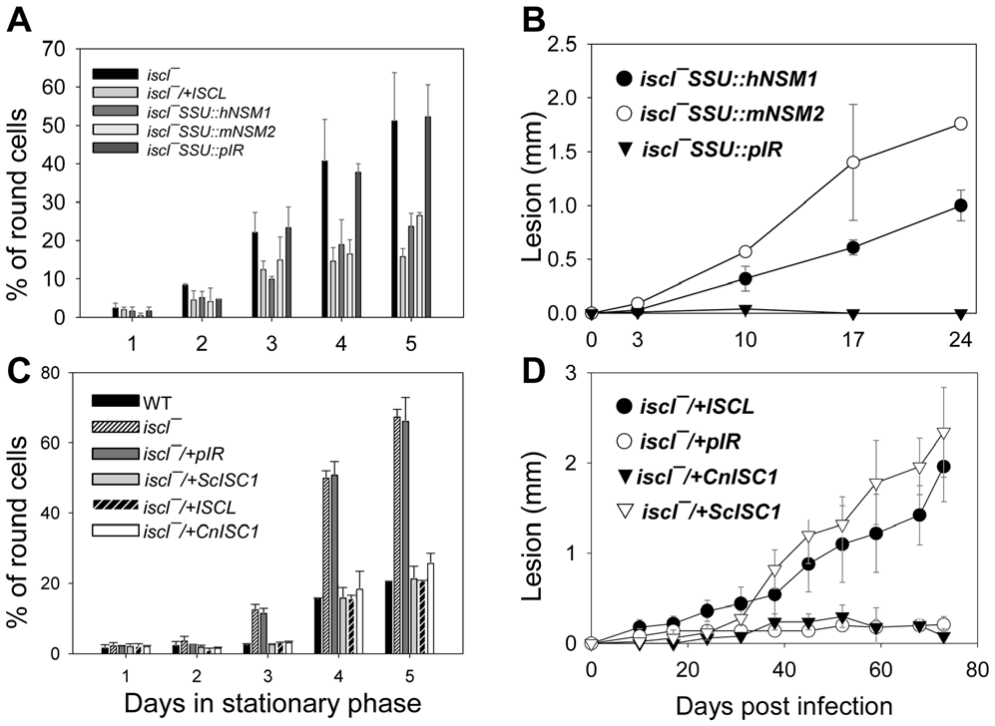
Restoration of stationary phase morphology and acute virulence in *iscl^−^* mutants by mammalian neutral SMases and fungal ISC1ps. (**A**)–(**B**) Morphology and virulence of *iscl^−^* parasites with human neutral SMase 1 (*iscl^−^* SSU::hNSM1), murine neutral SMase 2 (*iscl^−^* SSU::mNSM2), or pIR1SAT vector only (*iscl^−^* SSU::pIR) were examined as described. (**C**)–(**D**) Similar experiments were performed using *iscl^−^* parasites transfected with pIR1SAT-*ScISC1* (*iscl^−^/+ScISC1*), pIR1SAT-*CnISC1* (*iscl^−^/+CnISC1*), and pIR1SAT vector only (*iscl^−^*/+pIR). In (**A**) and (**C**), promastigotes were grown to stationary phase and the percentage of round cells (day 1 through day 5 in stationary phase) was determined. In (**B**) and (**D**), infectivity of stationary phase promastigotes was examined by footpad infection assay using BALB/c mice (2×10^7^ cells/mouse in **B** and 1×10^6^ cells in **D**) and the progression of lesions was monitored weekly. Error bars represent standard deviations.

Next we examined the effects of mammalian neutral SMases and ScISC1 on cell morphology and virulence in *iscl^−^*. As shown in Fig. 6A and 6B, both human neutral SMase 1 and murine neutral SMase 2 reversed the cell shape defects in *iscl^−^* during stationary phase and restored the ability to elicit lesion pathology in BALB/c mice (lesion sizes correlated with parasite numbers in the footpads, Table S1), whereas control parasites with the empty vector (*iscl^−^* SSU::pIR) behaved similarly to the parental *iscl^−^* mutants. Further analyses confirmed that *iscl^−^* SSU::hNSM1 and *iscl^−^* SSU::mNSM2 also had improved viability in late stationary phase (by propidium exclusion flow cytometry) and increased virulence in macrophage infections (data not shown). Therefore, defects in *iscl^−^* can be complemented by mammalian neutral SMases. Similar to mammalian enzymes, *ScISC1* completely restored morphology, viability, and virulence in *iscl^−^* parasites (Fig. 6C and 6D), indicating it could functionally substitute *ISCL*. Again, lesion pathology induced by *iscl^−^*/+*ScISC1* was consistent with parasite numbers over time (Table S2). Together, our complementation study strongly suggests that SMase activity is required for acute virulence in *L. major*.

### Expression of a specific IPCase fails to rescue *iscl^−^*

Recently, it was shown by the group of Maurizio Del Poeta (Medical University of South Carolina, Charleston) that unlike the ScISC1p, the ISC1 of *Cryptococcus neoformans* lacked significant activity with sphingomyelin while retaining activity with IPC (M. Del Poeta, personal communication). Heterologous expression of an enzyme lacking SMase activity could thus serve as a probe to test the relative importance of SMase vs. IPCase activity. The *C.neo* ISC1 ORF was inserted into pXG and introduced into the *iscl^−^* mutant, yielding *iscl^−^*/+*CnISC1*. Consistent with Del Poeta's findings, lysate from this line showed a high level of IPCase activity but little if any SMase activity (Fig. 5C–F; Table 1).

In contrast to the ability of the SL hydrolases with significant SMase activity, expression of *CnISC1* in *iscl^−^* did not restore their ability to induce lesion pathology in susceptible BALB/c mice infection (Fig. 6C–D). Instead, these *iscl^−^*/+*CnISC1* parasites persisted at low levels in BALB/c mice, similar to those *iscl^−^* mutants transfected with pIR1SAT vector only (Fig. 6C–D) (Table S2). Interestingly, the *iscl^−^*/+*CnISC1* parasites exhibited normal morphology (Fig. 6C) and viability (data not shown) in stationary phase. Therefore, the cell shape defect appears to be separate from the virulence defect in *iscl^−^*. In total, our complementation experiments with SL hydrolases suggest the degradation of host sphingomyelin is essential for acute pathology in *L. major*, whereas the activity of IPCase is not required for virulence (Table 1 and Fig. 6).

### Expression of surface virulence factors is unaltered in *iscl^−^* mutants

**Figure 7:**
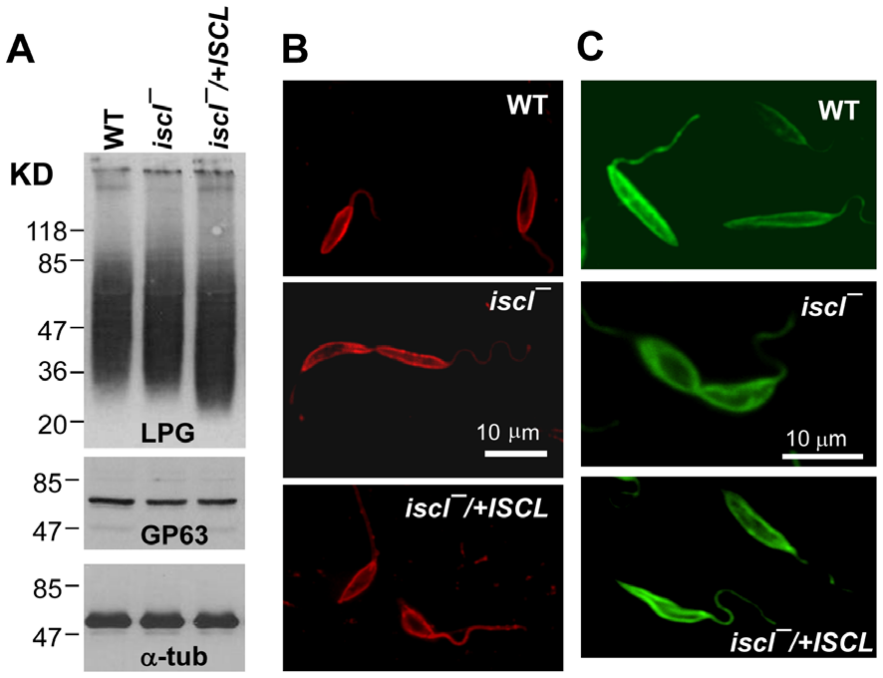
Expression of LPG and GP63 is not altered in *iscl^−^* mutants. (**A**) Whole cell lysates from log phase promastigotes were resolved by SDS/PAGE and blotted to PVDF membranes. Monoclonal antibody WIC79.3 was used to detect LPG; rabbit anti-*L. major* GP63 polyclonal antibody was used to detect GP63; and monoclonal anti-α-tubulin antibody (Sigma) was used as a loading control [41]. Localizations of LPG (**B**; primary antibody: WIC79.3; secondary antibody: goat anti-mouse IgG-Texas Red) and GP63 (**C**; primary antibody: a monoclonal anti-*L. major* GP63 antibody from Cedarlane Inc.; secondary antibody: goat anti-mouse IgG-FITC) were determined by indirect immuno-fluorescence microscopy as we previously described [12].

Surface glycoconjugates including LPG, GP63, and GIPLs are important virulence factors in *Leishmania*. Because the degradation of IPC generates phosphoinositol (Fig. S2), which could be used to synthesize GPI-anchored molecules, we tested whether the deletion of *ISCL* affects the production of LPG or GP63. Whole cell extracts from log phase promastigotes were subjected to western-blot analysis using monoclonal antibody WIC79.3 [41],[42] and a rabbit anti-GP63 antiserum to detect LPG and GP63, respectively. As illustrated in Fig. 7A, cellular levels of LPG and GP63 were not significantly altered in *iscl^−^* mutants or the *iscl^−^*/+*ISCL* parasites; in addition, both LPG and GP63 showed normal surface (cell membrane) localization in log and stationary phases (Fig. 7B and 7C; data not shown). Together, these results indicate the loss of *ISCL* has no adverse effects on the expression or localization of GPI-anchored molecules. Therefore, the loss of acute virulence in *iscl^−^* is not due to defects in surface virulent factors.

### Localization of ISCL

**Figure 8:**
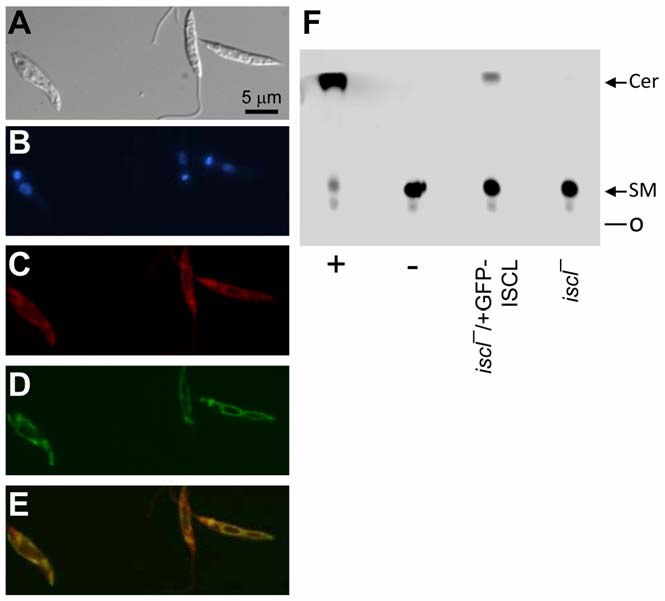
Cellular localization of GFP-tagged ISCL. (**A**–**E**) Log phase WT [pXG-*GFP-ISCL*] parasites were stained with MitoTracker Red580 and subjected to fluorescence microscopy. (**A**) DIC; (**B**) Hoechst staining; (**C**) MitoTracker staining; (**D**) GFP fluorescence; (**E**) Merge of **C** and **D**. (**F**) GFP-ISCL is functional. Whole cell lysates from *iscl^−^* or *iscl^−^* transfected with pXG-*GFP-ISCL* were tested for neutral SMase activity as described and a representative TLC image is shown. Positive control (+): 0.1 unit of *B. cereus* SMase; negative control (−): boiled WT lysate.

To determine the cellular localization of ISCL, a GFP-ISCL fusion protein was introduced into the *iscl^−^* mutant. Fluorescence microscopy revealed the distribution of GFP-ISCL to be similar to the staining pattern of the mitochondrial marker MitoTracker, in both log and stationary phase (Fig. 8A–E and data not shown), suggesting this protein is mostly localized in the mitochondria. The GFP-ISCL was functional, as *iscl^−^/+GFP-ISCL* parasites were able to degrade sphingomyelin (Fig. 8F) and showed normal morphology and virulence (data not shown). GFP-ISCL did not overlap with the plasma membrane, as revealed using an anti-LPG monoclonal antibody WIC 79.3 or the endoplasmic reticulum (ER) revealed by fluorescence microscopy with an anti-*T. brucei* Bip antiserum (data not shown).

Next, we examined whether ISCL could be secreted. Promastigotes of *iscl^−^/+ISCL* were grown from log to stationary phase and culture supernatant was examined for neutral SMase activity. As shown in Fig. S5, contrary to the robust SMase activity from *iscl^−^/+ISCL* cells, no activity was detected in the supernatant. Adding sphingomyelin to the culture medium did not induce any ISCL secretion (Fig. S5). Similar results were observed with concentrated supernatant (using filtration-based concentrators) (data not shown). Therefore, consistent with the localization study and the predicted ISCL sequence which lacks an obvious signal peptide, promastigotes do not secrete ISCL.

### Neutral SMase activity in *L. amazonensis* amastigotes

**Figure 9:**
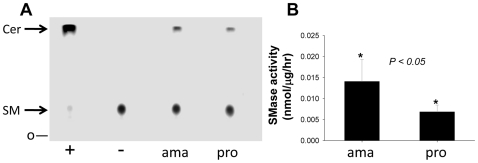
Detection of neutral SMase activity in *L. amazonensis* promastigotes and axenic amastigotes. Whole cell lysate from *L. amazonensis* promastigotes (pro) and axenic amastigotes (ama) were incubated with NBD-labeled C6 sphingomyelin and neutral SMase activity was determined by TLC (**A**) and normalized to nmol/µg/hour (**B**). Positive control (+): 0.1 unit of *B. cereus* SMase; negative control (−): boiled *L. major* WT lysate. Experiments were repeated multiple times and error bars represent standard deviations. *, *p*<0.05.

The lack of acute virulence in *iscl^−^* mutants suggests SMase activity is essential for amastigote survival and replication in the mammalian host. However, methods for the generation of axenic *L. major* parasites amastigotes are not available, and lesion-derived or macrophage-derived amastigotes typically are contaminated with host material likely including neutral SMase. To circumvent this problem, we used *L. amazonensis*, a new world *Leishmania* species able to form axenic amastigotes that resemble closely to lesion-derived amastigotes in morphology, virulence, expression of stage-specific genes, and interaction with mammalian cells [43],[44]. As shown in Fig. 9, lysates from both promastigotes and amastigotes of *L. amazonensis* were able to hydrolyze sphingomyelin. Similar to *L. major*, the neutral SMase activity in *L. amazonensis* promastigotes and amastigotes was sensitive to GW4869 (data not shown). After normalizing to protein levels, a two-fold higher level of activity was seen in amastigotes than promastigotes (Fig. 9) (*p*<0.05). Thus *L. amazonensis* amastigotes express SMase which was shown genetically in *L. major* to be required for parasite survival and growth in the mammalian host.

## Discussion

### *ISCL* is responsible for the degradation of host-derived sphingomyelin and *Leishmania*-derived IPC, but is not required for the production of EtN

In *L. major*, *ISCL* is the sole homologue of the neutral SMase genes in mammals and the *ISC1* genes (which encode **I**nositol phospho**S**phingolipid phospholipase **C** or IPCase) in fungi (Figs. S2 and S3). The predicted ISCL contains several well-conserved regions that are essential for the catalytic activity of SMase and ISC1p, e.g. a P-loop motif which may be involved in Mg^2+^ binding, and two transmembrane helices near the C-terminus, which may anchor the protein to mitochondrial membrane for activation [20],[27]. Despite the lack of sphingomyelin synthesis, *L. major* parasites can actively take up and hydrolyze sphingomyelin and the neutral SMase activity clearly requires *ISCL* (Fig. 3A–D). In addition to sphingomyelin, ISCL also contributes to the turnover of endogenous IPC as the *iscl^−^/+ISCL* parasites exhibited elevated IPCase activity whereas the activity in *iscl^−^* mutants was close to background (Fig. 3E–F). Consistent with this result, *iscl^−^* mutants showed modest accumulation of IPC and less ceramide compared to WT and *iscl^−^/+ISCL* parasites (Table 2). Similar to mammalian neutral SMases, *L. major* ISCL exhibited much stronger activity with sphingomyelin than IPC (10–20 times higher, Table 1), suggesting sphingomyelin is the preferred substrate.

For promastigotes, the synthesis and degradation of sphingoid base is a major pathway to generate EtN [14] (Fig. S1). Although *Leishmania* do possess other pathways to generate EtN (such as salvage from the medium), they are not sufficient to support growth and metacyclogenesis in the absence of SL metabolism as manifested by the *spt2^−^* and *spl^−^* mutants [14]. As illustrated in Fig. S1, the production of phosphoethanolamine from SL metabolites could occur via two routes: an IPC independent route from sphingoid bases and ceramide; and an alternative route which requires the synthesis and degradation of IPC (Fig. S1). The latter is considered a reasonable pathway because SL metabolites such as sphingoid bases and ceramides are toxic and labile with low steady state concentrations, whereas IPC is highly abundance and could serve as a reservoir for EtN production [14]. However, our results clearly indicate although ISCL is responsible for the degradation of IPC, it is not essential for EtN production. First, *iscl^−^* mutants are fundamentally different from *spt2^−^* and *spl^−^* mutants: *iscl^−^* parasites grow like WT *in vitro* and form apparently normal metacyclics yet fail to induce pathology in mice, whereas *spt2^−^* and *spl^−^* mutants grow well but upon entry into stationary phase die rapidly and fail to differentiate; however, they retain the ability to yield acute disease pathology, as some surviving parasites are able to differentiate into amastigotes, which are able to acquire ethanolamine by savage [14]; second, defects in *iscl^−^* mutants cannot be reversed by exogenous EtN at all (Figs. 1–2, 4); third, the abundance of PLE was similar between *iscl^−^* mutants and WT parasites (Table 2). Therefore, there is no shortage of EtN in *iscl^−^* mutants, suggesting the IPC-independent pathway is sufficient for EtN/PLE biosynthesis in the absence of ISCL (Fig. S1).

### The activity of neutral SMase but not IPCase is essential for parasite proliferation and pathology in mammals

The most striking defect of *iscl^−^* mutants is their complete lack of acute virulence in susceptible BALB/c mice, as stationary phase promastigotes or purified metacyclics yielded no pathology even after 7–8 months (Fig. 4A–D). *In vitro* macrophage infection indicated that *iscl^−^* were able to enter host cells but did not survive well, especially at 37 °C (Fig. 4E–H). This virulence defect was completely reversed by the neutral SMases from human, mouse, and yeast (Table 1) (Fig. 6). We confirmed the finding from Del Poeta's group that the *C. neo* ISC1 lacked significant SMase activity in our studies, when it was expressed heterologously in *Leishmania* (Fig. 5). Despite its strong IPCase activity, heterologous expression of *CnISC1* failed to reverse the virulence defect (Figs. 5–6). Together, these complementation results strongly suggest the degradation of host-derived sphingomyelin is necessary and sufficient for parasite survival and replication in mammals, whereas the degradation of endogenous IPC by itself is not essential for *Leishmania* virulence. In agreement with this conclusion, axenic amastigotes of *L. amazonensis* showed strong SMase activity, about 2-fold higher than promastigotes (Fig. 9).

Interestingly, most GFP-tagged ISCL is localized in the mitochondria during the promastigote stage (Fig. 8) when cells can take in sphingomyelin for hydrolysis (Fig. 3E–F). In *Leishmania*, lipid vesicles and lipid-protein complexes can be incorporated into the parasite plasma membrane through fusion or be taken up through an endocytic pathway including the flagellar pocket and endosomes [45],[46]. Although mitochondria are not directly connected to the vesicular pathways, phospholipids such as PtS are synthesized in the ER and transported to the mitochondria transported in mammalian cells and yeast [47],[48]. In *S. cerevisiae*, an ER-mitochondria tethering complex has been identified composed of proteins resident of both ER and mitochondria [49]. Consistent with these findings, our data imply lipids from the plasma membrane and/or endocytic compartments may contribute to the homeostasis of mitochondrial membrane (where GFP-ISCL resides and the hydrolysis of SLs occurs). Localization of ISCL in amastigotes is currently under investigation.

In *S. cerevisiae*, ScISC1p generates phytoceramide in the mitochondrial membrane and such activity may be important for the respiratory function of mitochondria and the metabolism of non-fermentable carbon sources [36]. As promastigotes, *Leishmania* acquire energy through the metabolism of sugars, amino acids and fatty acids [50]. It is possible that the mitochondrion-localized ISCL has similar functions as the *Sc* enzyme in regulating respiration and plays an important role in parasite growth in low sugar conditions. Consistent with this hypothesis, *iscl^−^* mutants showed poor viability and were more round in shape (signs of cells under stress) in late stationary phase when glucose became depleted (Fig. 1). Interestingly, although CnISC1p failed to complement the virulence defect in *iscl^−^*, it did restore the mutants' morphology and viability in late stationary phase, implying that while SMase activity is required for the survival and proliferation of amastigotes, IPC hydrolysis, however, may be involved in the maintenance of mitochondrial function in promastigotes.

In addition to its potential role in mitochondria, we could envision several other mechanisms by which SMase activity may contribute to virulence; notably these mechanisms are not mutually exclusively. First, amastigotes of most *Leishmania* species reside within mature phagolysosomes which are enriched in amino acids and lipids but poor in carbohydrates [51]. To survive, these amastigotes need to salvage lipids (including SLs) from the host [15] and SMase (along with other lipases) may be required for the acquisition of nutrients. In addition, the degradation of host SLs by amastigotes could disrupt SL-dependent signaling pathways in macrophages. It has been reported that *Leishmania donovani* infection led to elevated ceramide levels in macrophages, which were responsible for the downregulation of classical PKC activity and the induction of PKCzeta (an atypical Ca-independent stress kinase), as well as the ceramide-activated protein phosphatases. These changes were associated with the inhibition of NF-kappaB transactivation and the suppression of nitric oxide generation [52]–[54]. In the future, molecular interactions between *iscl^−^* mutants and host phagocytes will be evaluated to determine whether sphingomyelin degradation is required for the inhibition of proinflammatory cytokine release. In addition, the ability of *iscl^−^* mutants to persist without pathology provides an excellent platform to study the long-term, asymptomatic infection where the interaction between *Leishmania* and host is poorly understood.

### Summary

Our study revealed an essential yet previously unrecognized role of SL degradation in *L. major* virulence. Despite the lack of sphingomyelin biosynthesis, *Leishmania* parasites possess an *ISCL*-dependent neutral SMase activity. Deletion of *ISCL* completely abolished acute disease pathology in mice. *ISCL* is also required for the turnover of IPC, but is not required for the production of EtN. Future studies will determine the molecular mechanism by which host-SL degradation contributes to acute virulence.

## Materials and Methods

### Materials

BALB/c (female, 7–8 weeks old) mice were purchased from Charles River Laboratories International. All procedures involving mice were approved by the Animal Care and Use Committee at Texas Tech University (PHS Approved Animal Welfare Assurance No. A3629-01).

N-[6-[(7-nitro-2-1,3-benzoxadiazol-4-yl)amino]hexanoyl]-sphingosine-1-phosphocholine (NBD C6-sphingomyelin) was purchased from Invitrogen Corporation. N-[12-[(7-nitro-2-1,3-benzoxadiazol-4-yl)amino]dodecanoyl]-sphingosine-1-phosphoinositol (NBD C12-IPC) was custom-synthesized by Avanti Polar lipids. All other chemicals were purchased from VWR International unless specified otherwise.

### Molecular constructs

The open reading frame (ORF) of *ISCL* (LmjF08.0200) was amplified by PCR from *L. major* genomic DNA using primer pairs 5′*ISCL* ORF BamHI (attactGGATCCACCATGTCGCACGCATCGACCTT, P58) and 3′*ISCL* ORF BamHI (attactGGATCCCTACAACTTCTTCAGCT, P59). The resulting DNA fragment was digested with *BamHI* and cloned in the pXG vector [55] as pXG-*ISCL* (B83) or the pIR1SAT vector [56] as pIR1SAT-*ISCL* (B106). After confirming its sequence, *ISCL* ORF was cloned into the pXG-GFP+2′ vector to generate pXG-GFP-*ISCL* (B103), which was used in localization studies. To generate the knock-out constructs for *ISCL*, the predicted 5′- and 3′-untranslated regions (∼1 Kb each) were PCR amplified and cloned in tandem in the pUC18 vector; genes conferring resistance to puromycin (*PAC*) and blasticidin (*BSD*) were inserted between the 5′- and 3′-untranslated regions to generate pUC-KO-*ISCL:PAC* (B85) and pUC-KO-*ISCL:BSD* (B84). ORFs of human neutral SMase 1 (accession #NP_003071) and mouse neutral SMase 2 (accession #NM_021491.3), derived from constructs generated in Dr. Yusuf Hannun's lab (Medical University of South Carolina), were cloned in the pIR1SAT vector as pIR1SAT-*hNSM1* (B142) and pIR1SAT-*mNSM2* (B141), respectively. *ISC1* genes from *S. cerevisiae* (*ScISC1*, accession #P40015) and *C. neoformans* (*CnISC1*, accession #DQ487762) were PCR amplified and cloned in the pIR1SAT vector as pIR1SAT-*ScISC1* (B108) and pIR1SAT-*CnISC1* (B107), respectively.

### *Leishmania* culture and genetic manipulations

*L. major* LV39 clone 5 (Rho/SU/59/P) promastigotes were grown in M199 medium with 10% fetal bovine serum and other supplements as described [57]. *L. amazonensis* (MHOM/BR/77/LTB0016) promastigotes and axenic amastigotes were cultured as previously described [43],[58]. Growth rate was determined by monitoring the density of culture over time using a hemacytometer. Cell viability was measured by flow cytometry using an Accuri C6 Flow Cytometer after staining with 5 µg/ml of propidium iodide. Metacyclics were isolated from stationary phase culture using the density gradient method [34] and/or the peanut agglutination method [33]. The *ISCL* alleles were sequentially replaced by puromycin (*PAC*) and blasticidin (*BSD*) resistance genes to generate the *iscl^−^* mutant (*ΔISCL::BSD/ΔISCL::PAC*). Transfection and selection of promastigotes were performed as previous described [57]. To confirm the deletion of *ISCL*, genomic DNA was digested with *NotI* plus *BamHI*, resolved on a 0.8% agarose gel, transferred to a nitrocellulose membrane, and hybridized with a [^32^P]-labeled DNA probe. To re-constitute *ISCL* expression, pXG-*ISCL* was introduced into *iscl^−^* and referred to as *iscl^−^/+ISCL* (*ΔISCL::BSD/ΔISCL::PAC*+ pXG-*ISCL*). To test whether *iscl^−^* can be complemented by mammalian neutral SMases, linearized DNA (using *SwaI*) from pIR1SAT-*hNSM1* or pIR1SAT-*mNSM2* were integrated into the small ribosomal subunit site of *iscl^−^* mutants to generate *iscl^−^* SSU::hNSM1 or *iscl^−^* SSU::mNSM2. To test whether *iscl^−^* can be complemented by fungal *ISC1* genes, mutants were transfected with pIR1SAT-*ScISC1* or pIR1SAT-*CnISC1* to generate *iscl^−^/+ScISC1* or *iscl^−^/+CnISC1*. To study the localization of GFP-tagged ISCL protein, WT parasites were transfected with pXG-GFP-*ISCL* and WT [pXG-GFP-*ISCL*] cells were selected based on resistance to G418.

### Microscopy and macrophage infection

To analyze the morphology of *iscl^−^* mutants, log and stationary phase promastigotes were fixed in 3.7% formaldehyde and percentages of round cells (defined as those with the length of the long axis less than twice the length of the short axis) were determined using a hemacytometer. About 200 randomly selected cells were counted in each experiment. For fluorescence microscopy, parasites were stained with 350 nM of Mitotracker Red 580 (Invitrogen) for 30min in darkness. Cells were then attached to poly-lysine coated cover slips, washed twice with phosphate buffered saline (PBS), once with 50% ethanol, and stained with 2.5 µg/ml of Hoechst 33342 for 10min. Images were acquired using an Olympus BX50 Upright Fluorescence Microscope equipped with a digital camera. Localizations of LPG (primary antibody: WIC79.3 [42]; secondary antibody: goat anti-mouse IgG-Texas Red) and GP63 (primary antibody: monoclonal anti-*L. major* GP63 antibody from Cedarlane Inc.; secondary antibody: goat anti-mouse IgG-FITC) were determined by indirect immuno-fluorescence microscopy as we previously described [12].

Peritoneal macrophages from BALB/c mice were isolated and infected with purified stationary phase promastigotes (opsonized with C57BL6 mouse serum) at a ratio of ten parasites per one macrophage as previously described [38]. Percentages of infected macrophages and the number of parasites per 100 macrophages were determined microscopically after staining with 2.5 µg/ml of Hoechst 33342.

### Mouse infections and analyses

Virulence of promastigotes was evaluated in BALB/c mice using footpad infection [59] and ear lobe infection [60],[61]. For footpad assay, late stationary phase promastigotes (3 days after the onset of stationary phase) or purified metacyclics (prepared from 3–4 days old stationary phase culture) were resuspended in DMEM and injected into the footpads of 8-week old female BALB/c mice (6–10 mice per group) at 1×10^6^ cells/mouse (stationary phase promastigotes) or 2×10^5^ cells/mouse (metacyclics). For ear lobe infection, stationary phase parasites were inoculated intradermally into the ear lobes of 8-week old female BALB/c mice (5 mice per group) at 1×10^5^ cells/mouse. Lesion sizes were measured weekly using a vernier caliper and parasite numbers in the infected tissue were determined by limiting dilution assay [59]. Mice infected with WT or *iscl^−^/+ISCL* parasites were sacrificed when their lesions became overly large (over 2.5 mm for footpad infection and over 5.0 mm for ear infection).

### Neutral SMase assay and IPCase assay

Log phase promastigotes were suspended in a lysis buffer (25mM Tris pH7.5, 0.1% Triton X100, 1× protease inhibitor) at 2.0×10^8^ cells/ml and incubated for 5 min on ice. Protein concentration was determined by the micro-BCA assay. Triton X100/lipid mixed micelles were prepared as previously described [29] with minor modifications. For neutral SMase assay, 40 µg of *Leishmania* protein (∼20 µl of lysate) was incubated in 100 µl of buffer containing 50mM Tris pH7.5, 5mM MgCl_2_, 5mM dithiothreitol, 0.1% Triton X100, 11 nmol of PtS (Avanti), 2.8 nmol of unlabeled sphingomyelin (Avanti), and 0.8 nmol of NBD C6-sphingomyelin. After incubation at room temperature for 60 min, 1 ml of chloroform, 0.5 ml of methanol, and 0.2 ml of water were added to each reaction and lipid was extracted, dried, and resuspended in 20 µl of chloroform: methanol (1∶2). Thin layer chromatography (TLC) was performed as we previously described [12] and plates were scanned with a Storm 860 phosphoimager. Results were normalized to nmol/µg/hour after subtracting the value of negative control. 0.1 unit of *Bacillus cereus* SMase (Sigma) was used as a positive control and boiled WT lysate (40 µg) was used as a negative control. For IPCase assay, similar experiments were performed except: 1) lysate was incubated in the absence of sphingomyelin and presence of 0.8 nmol of NBD C12-IPC; 2) TLC plates were developed in a different solvent (chloroform∶methanol∶water = 65∶24∶5); and 3) 0.1 unit of *Bacillus cereus* phosphatidylinositol phospholipase C (PI-PLC, Sigma) was used as a positive control.

### Degradation of sphingomyelin by intact *L. major* promastigotes

Promastigotes were inoculated in M199 medium without fetal bovine serum at 7.0×10^5^ cells/ml and labeled with 5 µM of NBD C6-sphingomyelin. After 48 h, cells were washed with PBS and total lipids were extracted and analyzed by TLC as described above for neutral SMase assay.

### Lipid analysis by electrospray ionization mass spectrometry

Lipid extraction and analysis by ESI/MS (negative ion mode) was performed as previously described [15] with minor modifications. A *N*-octanoyl-D-*erythro*-sphingosine-1-phosphate (d18:1/8:0 ceramide phosphate, FW = 505.5, 1.0×10^8^ molecules/cell) was used as a standard for IPC; a 1-*O*-1′-(Z)-octadecenyl-2-arachidoyl-*sn*-glycero-3-phosphoethanolamine (*p*18:0/20:4-PE, FW = 751.6, 2.0×10^8^ molecules/cell) was used as a standard for PLE; and a *N*-octanoyl-D-*erythro*-sphingosine (d18:1/8:0 ceramide, FW = 425.7, 1.0×10^8^ molecules/cell) was used as a standard for ceramide. All three internal standards were added prior to lipid extraction.

### Western-blot of LPG and GP63

Promastigotes were collected and resuspended in PBS at 1×10^8^cells/ml. Cell extracts were prepared and western-blotting were performed as previously described [12]. Primary antibodies include the rabbit anti-GP63 polyclonal antiserum (a generous gift from Dr. KP Chang at Rosalind Franklin University of Medicine and Science) (1∶10000), monoclonal anti-*L. major* LPG antibody WIC79.3 [42] (1∶5000), and monoclonal anti-α-tubulin antibody (Sigma) (1∶8000); secondary antibodies include the goat anti-rabbit or anti-mouse IgG Ab–HRP conjugated (1∶2000).

## Supporting Information

Figure S1 Metabolism of SLs in *L. major*. SPT: serine palmitoyltransferase; SPL: sphingosine- 1-phosphate lyase; ISC1p: inositol phosphosphingolipid phospholipase C 1 protein or IPCase; IPCS: IPC synthase. Note that phosphoethanolamine can be produced via an IPC-independent pathway or an indirect pathway that requires the synthesis and degradation of IPC.
(0.07 MB PDF)

Figure S2 Degradation of sphingomyelin (A) and IPC (B). In mammals, the degradation of sphingomyelin by SMase is a major route to produce ceramide, an important signaling molecule. *Leishmania* parasites do not synthesize sphingomyelin but contain high abundance of IPC. In fungi (which also synthesize IPC), hydrolysis of IPC is mediated by inositol phosphosphingolipid phospholipase C (ISC1p, B), a homolog of mammalian neutral SMase.
(0.11 MB PDF)

Figure S3 Alignment of the amino acid sequences of LmISCLp (*L. major* geneDB system ID LmjF08.0200), human neutral SMase 1 (Genbank accession #NP_003071), and *Saccharomyces cerevisiae* ISC1p (Genbank accession #P40015). Highly conserved regions are shaded. The P-loop domain is underlined. The two boxed regions near the C-terminus of *Lm*ISCLp (at aa 447–466 and 612–634) represent predicted transmembrane helices. Asterisks represent amino acids essential for catalysis based on studies of *B. cereus* SMase [26], *Sc*ISC1p [27], and *Hs*NSMase 1 [28].
(0.11 MB PDF)

Figure S4 Targeted deletion of ISCL. Southern blot analysis of *L. major* wild type (WT), heterozygote clones (*ISCL*+/− #1 and 2), homozygote clones (*iscl^−^* #1-1 and #2-1), and the reconstituted strain (*iscl^−^*/+*ISCL*) was performed as described in *Materials and Methods* using a probe corresponding to the *ISCL* ORF. Bands corresponding to the endogenous (>10 Kb, 24 hours exposure) and episomal alleles of *ISCL* (∼2.0 Kb, 3 hours exposure) are indicated.
(0.05 MB PDF)

Figure S5 *L. major* promastigotes do not secrete ISCL protein. Promastigotes of *iscl^−^*/+*ISCL* were grown in the absence or presence of C16-sphingomyelin (provided at 1 µM final concentration daily) and neutral SMase assay was performed using whole cell lysate or culture supernatant. Positive control (+): 0.1 unit of *B. cereus* SMase; negative control (−): boiled WT lysate.
(0.06 MB PDF)

Table S1 Parasite number in BALB/c mice infected with *iscl^−^SSU::hNSM1*, *iscl^−^SSU::mNSM2*, and *iscl^−^SSU::pIR* (as described in Fig. 6B). Limiting dilution assays were performed at 4–5 weeks post infection (two mice per group).
(0.01 MB PDF)

Table S2 Parasite numbers in BALB/c mice infected with *L. major* promastigote (as described in Fig. 6D). Limiting dilution assays were performed at 6–7 weeks post infection (two mice per group).
(0.01 MB PDF)
