# Supplementary figures and images for: Degradation of Host Sphingomyelin Is Essential for Leishmania Virulence
Source: PLoS Pathog. 2009 Dec 11;5(12):e1000692. doi: 10.1371/journal.ppat.1000692 (PMC2784226; doi:10.1371/journal.ppat.1000692)

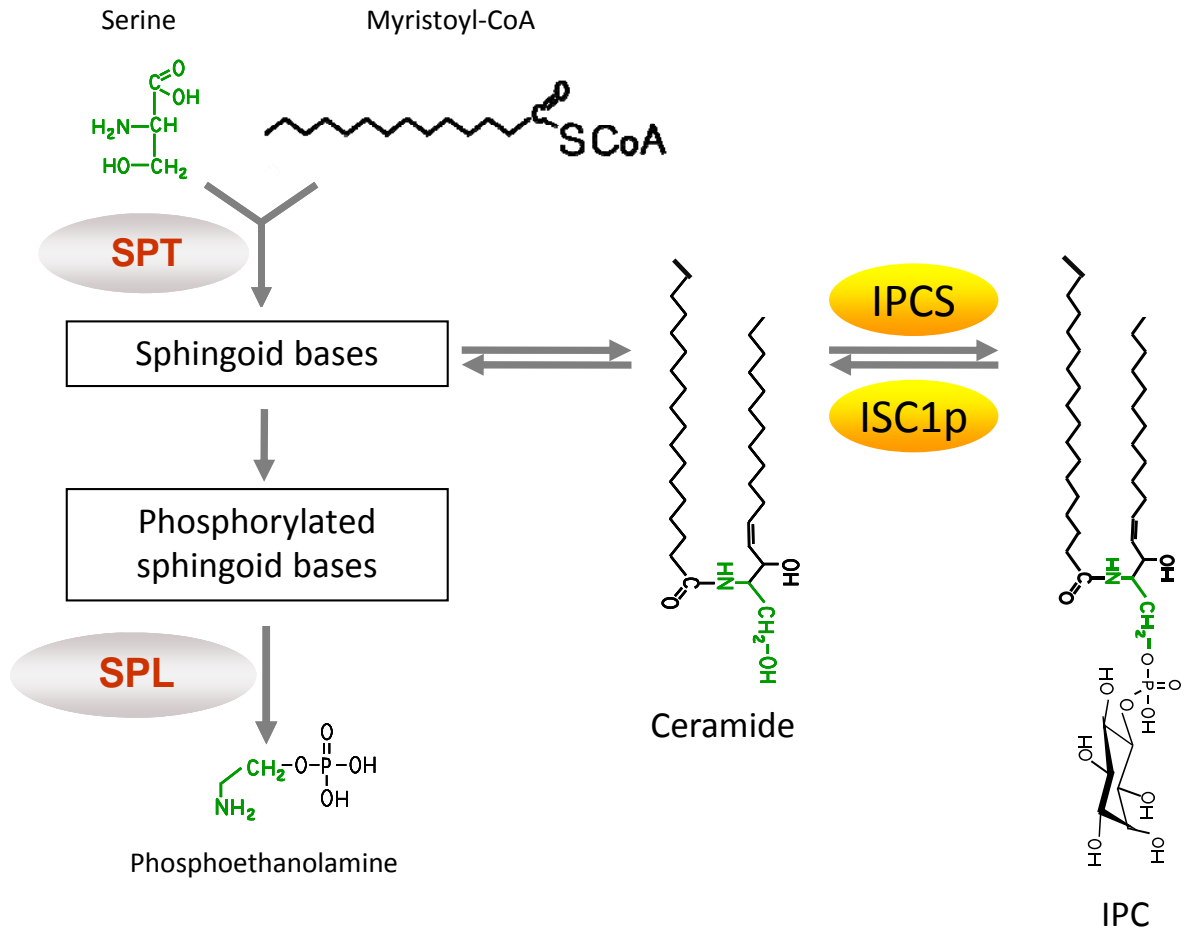

Supplement: Figure S1 — Metabolism of SLs in L. major. SPT: serine palmitoyltransferase; SPL: sphingosine- 1-phosphate lyase; ISC1p: inositol phosphosphingolipid phospholipase C 1 protein or IPCase; IPCS: IPC synthase. Note that phosphoethanolamine can be produced via an IPC-independent pathway or an indirect pathway that requires the synthesis and degradation of IPC. (0.07 MB PDF) [file ppat.1000692.s001.pdf]

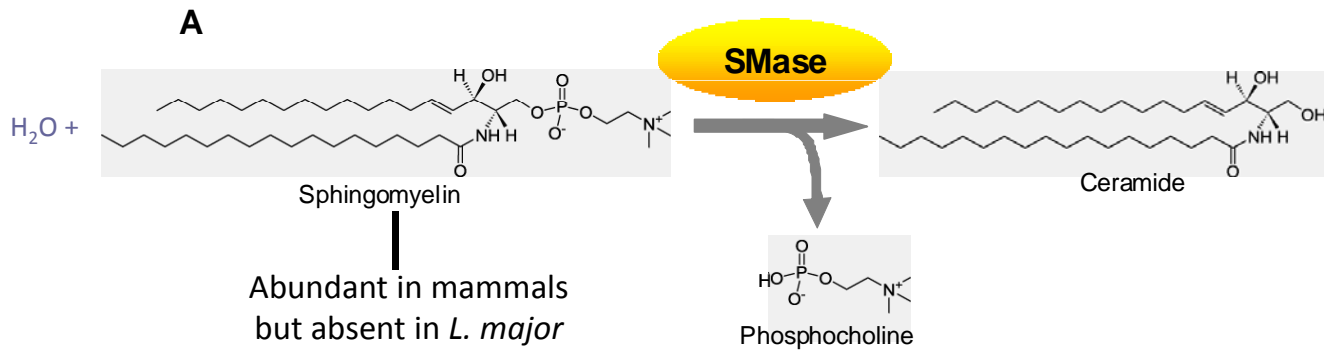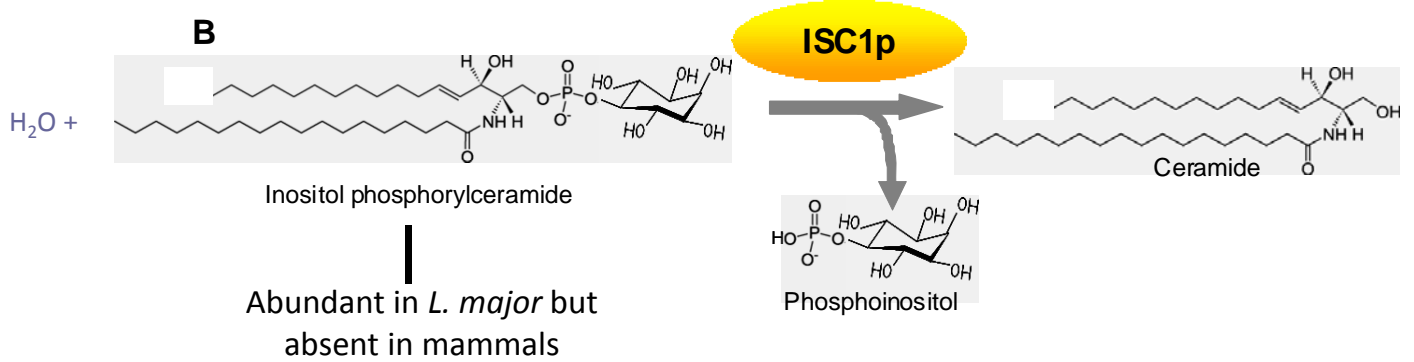

Supplement: Figure S2 — Degradation of sphingomyelin (A) and IPC (B). In mammals, the degradation of sphingomyelin by SMase is a major route to produce ceramide, an important signaling molecule. Leishmania parasites do not synthesize sphingomyelin but contain high abundance of IPC. In fungi (which also synthesize IPC), hydrolysis of IPC is mediated by inositol phosphosphingolipid phospholipase C (ISC1p, B), a homolog of mammalian neutral SMase. (0.11 MB PDF) [file ppat.1000692.s002.pdf]

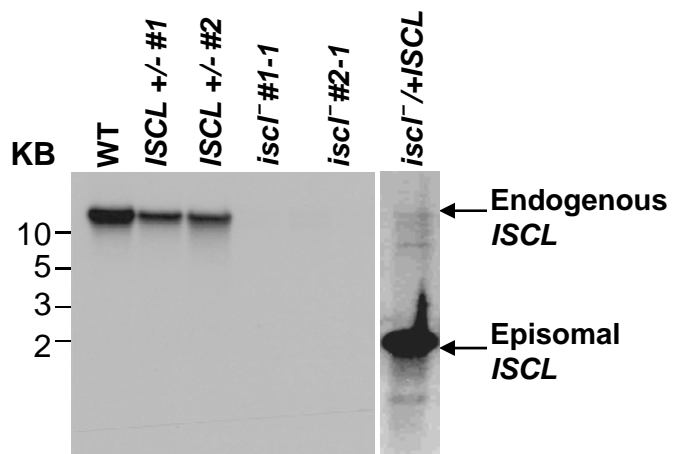

Supplement: Figure S4 — Targeted deletion of ISCL. Southern blot analysis of L. major wild type (WT), heterozygote clones (ISCL+/− #1 and 2), homozygote clones (iscl− #1-1 and #2-1), and the reconstituted strain (iscl−/+ISCL) was performed as described in Materials and Methods using a probe corresponding to the ISCL ORF. Bands corresponding to the endogenous (>10 Kb, 24 hours exposure) and episomal alleles of ISCL (∼2.0 Kb, 3 hours exposure) are indicated. (0.05 MB PDF) [file ppat.1000692.s004.pdf]

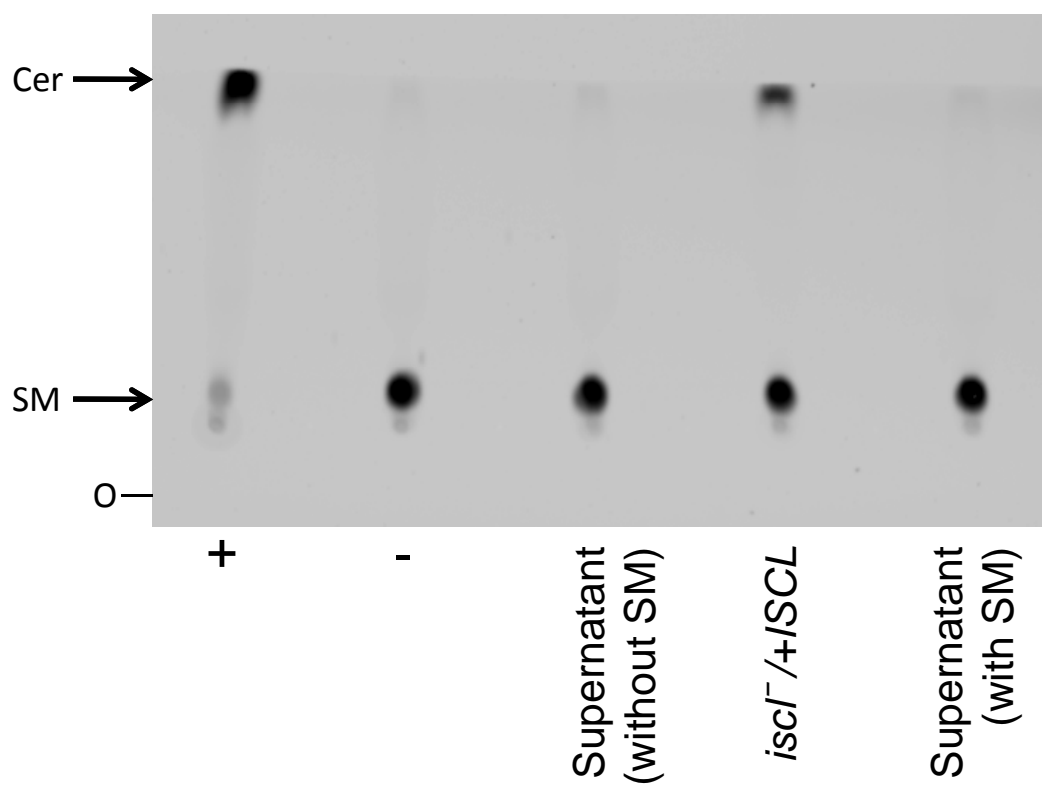

Supplement: Figure S5 — L. major promastigotes do not secrete ISCL protein. Promastigotes of iscl−/+ISCL were grown in the absence or presence of C16-sphingomyelin (provided at 1 µM final concentration daily) and neutral SMase assay was performed using whole cell lysate or culture supernatant. Positive control (+): 0.1 unit of B. cereus SMase; negative control (−): boiled WT lysate. (0.06 MB PDF) [file ppat.1000692.s005.pdf]
