# Supplementary material for: Degradation of Host Sphingomyelin Is Essential for Leishmania Virulence
Source: PLoS Pathog. 2009 Dec 11;5(12):e1000692. doi: 10.1371/journal.ppat.1000692 (PMC2784226; doi:10.1371/journal.ppat.1000692)
Supplement: Figure S3 — Alignment of the amino acid sequences of LmISCLp (L. major geneDB system ID LmjF08.0200), human neutral SMase 1 (Genbank accession #NP_003071), and Saccharomyces cerevisiae ISC1p (Genbank accession #P40015). Highly conserved regions are shaded. The P-loop domain is underlined. The two boxed regions near the C-terminus of LmISCLp (at aa 447–466 and 612–634) represent predicted transmembrane helices. Asterisks represent amino acids essential for catalysis based on studies of B. cereus SMase [26], ScISC1p [27], and HsNSMase 1 [28]. (0.11 MB PDF) [file ppat.1000692.s003.pdf]

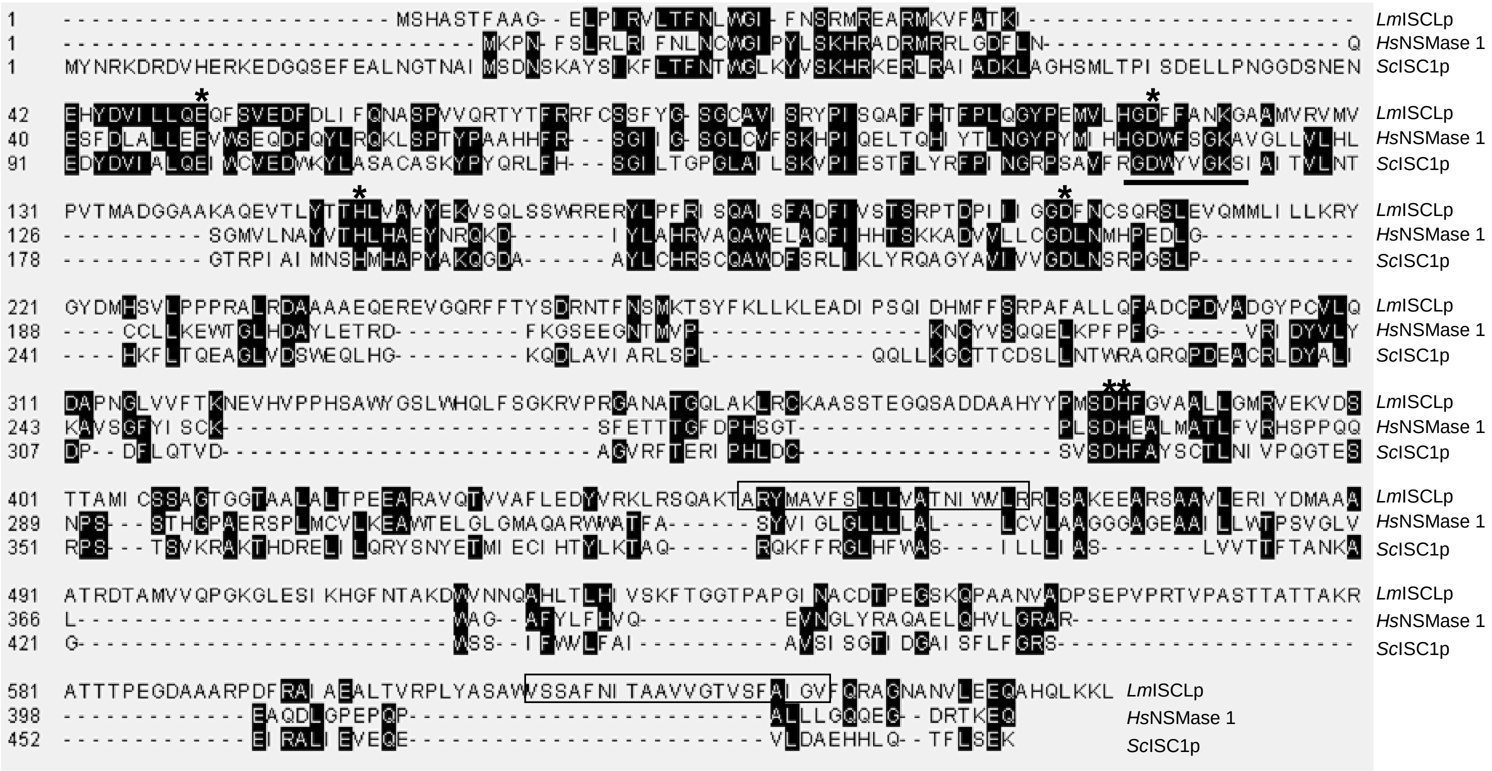
LmISCLp
HsNSMase 1
ScISC1p
