# Supplementary material for: Degradation of Host Sphingomyelin Is Essential for Leishmania Virulence
Source: PLoS Pathog. 2009 Dec 11;5(12):e1000692. doi: 10.1371/journal.ppat.1000692 (PMC2784226; doi:10.1371/journal.ppat.1000692)
Supplement: Table S1 — Parasite number in BALB/c mice infected with iscl−SSU::hNSM1, iscl−SSU::mNSM2, and iscl−SSU::pIR (as described in Fig. 6B). Limiting dilution assays were performed at 4–5 weeks post infection (two mice per group). (0.01 MB PDF) [file ppat.1000692.s006.pdf]

Table S1. Parasite number in BALB/c mice infected with *iscl¯SSU::hNSM1*, *iscl¯SSU::mNSM2*, and *iscl¯SSU::pIR* (as described in Fig. 6B).

	iscl¯SSU::hNSM1	*iscl¯SSU::mNSM2*	*iscl¯SSU::pIR*
Mouse #1	1.40×10^{6}	1.50×10^{7}	56
Mouse # 2	1.90×10^{6}	1.15×10^{7}	42
Average	1.65×10^{6}	1.33×10^{7}	49

Limiting dilution assays were performed at 4-5 weeks post infection (two mice per group).
