# Supplementary material for: Degradation of Host Sphingomyelin Is Essential for Leishmania Virulence
Source: PLoS Pathog. 2009 Dec 11;5(12):e1000692. doi: 10.1371/journal.ppat.1000692 (PMC2784226; doi:10.1371/journal.ppat.1000692)
Supplement: Table S2 — Parasite numbers in BALB/c mice infected with L. major promastigote (as described in Fig. 6D). Limiting dilution assays were performed at 6–7 weeks post infection (two mice per group). (0.01 MB PDF) [file ppat.1000692.s007.pdf]

Table S2. Parasite number in BALB/c mice infected with *L. major* promastigote (as described in Fig. 6D).

	iscl^{-}*/+pIR*	*iscl*$^{-}$*/+ISCL*	*iscl*$^{-}$*/+ ScISC1*	*iscl*$^{-}$*/+ CnISC1*
Mouse #1	42	2.07×10^{7}	1.51×10^{8}	28
Mouse # 2	28	8.60×10^{7}	1.06×10^{8}	56
Average	35	5.34×10^{7}	1.29×10^{8}	42

Limiting dilution assays were performed at 6-7 weeks post infection (two mice per group).
